# Perspectives of clients and providers on factors influencing opioid agonist treatment uptake among HIV-positive people who use drugs in Indonesia, Ukraine, and Vietnam: HPTN 074 study

**DOI:** 10.1186/s12954-020-00415-x

**Published:** 2020-10-01

**Authors:** Tetiana Kiriazova, Vivian F. Go, Rebecca B. Hershow, Erica L. Hamilton, Riza Sarasvita, Quynh Bui, Kathryn E. Lancaster, Kostyantyn Dumchev, Irving F. Hoffman, William C. Miller, Carl A. Latkin

**Affiliations:** 1 Ukrainian Institute On Public Health Policy, 5 Biloruska Str., Office 20, 27, Kyiv, 04050 Ukraine; 2 Department of Health Behavior, Gillings School of Global Public Health, The University of North Carolina At Chapel Hill, 135 Dauer Drive, Chapel Hill, NC 27599 USA; 3 Science Facilitation Department, FHI 360, 359 Blackwell Street, Suite 200, Durham, NC 27701 USA; 4 Dr. Cipto Mangunkusumo National Central General Hospital, University of Indonesia, Jalan Pangeran Diponegoro No.71, Salemba, Senen, Jakarta Pusat, Daerah Khusus Ibukota, Jakarta, 10430 Indonesia; 5 UNC Project Vietnam, Yen Hoa Health Clinic, Lot E2, Duong Dinh Nghe Street, Cau Giay District, Hanoi, Vietnam; 6 Division of Epidemiology, College of Public Health, The Ohio State University, 300-D Cunz Hall, 1841 Neil Avenue, Columbus, OH 43210 USA; 7 Division of Infectious Diseases, School of Medicine, The University of North Carolina At Chapel Hill, 130 Mason Farm Rd, Chapel Hill, NC 27599 USA; 8 Department of Health, Behavior, and Society, Johns Hopkins University, 615 N. Wolfe Street, Baltimore, MD 21205 USA

**Keywords:** People who inject drugs, Barriers to care, Opioid agonist treatment (OAT), Drug treatment, Indonesia, Ukraine, Vietnam, In-depth interviews

## Abstract

**Background:**

Opioid agonist treatment (OAT) is an effective method of addiction treatment and HIV prevention. However, globally, people who inject drugs (PWID) have insufficient OAT uptake. To expand OAT access and uptake, policymakers, program developers and healthcare providers should be aware of barriers to and facilitators of OAT uptake among PWID.

**Methods:**

As a part of the HPTN 074 study, which assessed the feasibility of an intervention to facilitate HIV treatment and OAT in PWID living with HIV in Indonesia, Ukraine, and Vietnam, we conducted in-depth interviews with 37 HIV-positive PWID and 25 healthcare providers to explore barriers to and facilitators of OAT uptake. All interviews were audio-recorded, transcribed, translated into English, and coded in NVivo for analysis. We developed matrices to identify emergent themes and patterns.

**Results:**

Despite some reported country-specific factors, PWID and healthcare providers at all geographic locations reported similar barriers to OAT initiation, such as complicated procedures to initiate OAT, problematic clinic access, lack of information on OAT, misconceptions about methadone, financial burden, and stigma toward PWID. However, while PWID reported fear of drug interaction (OAT and antiretroviral therapy), providers perceived that PWID prioritized drug use over caring for their health and hence were less motivated to take up ART and OAT. Motivation for a life change and social support were reported to be facilitators.

**Conclusion:**

These results highlight a need for support for PWID to initiate and retain in drug treatment. To expand OAT in all three countries, it is necessary to facilitate access and ensure low-threshold, financially affordable OAT programs for PWID, accompanied with supporting interventions. PWID attitudes and beliefs about OAT indicate the need for informational campaigns to counter misinformation and stigma associated with addiction and OAT (especially methadone).

## Introduction

Opioid agonist treatment (OAT) is an effective HIV prevention and treatment engagement strategy for opioid-dependent people who inject drugs (PWID) in Eastern Europe and Southeast Asia, where the HIV epidemic is still significantly driven by injection drug use [1, 2]. Globally, OAT improves health, reduces comorbidity and mortality [3, 4], promotes access to health care, and enhances quality of life [5, 6]. OAT also reduces risks of HIV transmission, overdoses, crime, and incarceration [7–9]. For PWID living with HIV, OAT improves linkage to HIV care, adherence to antiretroviral therapy (ART), and HIV treatment outcomes [10–15]. Integrated provision of OAT and HIV care was found beneficial for both treatments in multiple settings and was recognized as a priority strategy by WHO [16].

Despite these well-documented benefits of OAT, poor scale-up, low coverage, and poor retention diminish its potential benefits. Coverage with OAT and needle and syringe programs (NSP) remains poor at the global level, especially in the regions with the largest populations of PWID (East and Southeast Asia, Eastern Europe, and North America) [17, 18]. Current OAT coverage is insufficient to impact the epidemics of HIV and hepatitis C among PWID in many countries [17, 18]. Although buprenorphine is also available, most programs in developing countries offer methadone maintenance therapy as the main drug and program for OAT.

PWID experience numerous multilevel barriers to enrollment and retention in OAT. These barriers include structural and institutional barriers (bureaucracy, complex entry process, financial and logistical barriers) [19–22], stigma toward PWID and OAT patients [23, 24], and misconceptions among patients and providers about benefits of methadone treatment [5, 19, 25]. Facilitators to OAT include psychological, social, and financial support, availability of integrated addiction and HIV treatment, and OAT education [26, 27]. Additionally, PWID engagement in OAT may be influenced by country-specific individual, institutional, structural, and policy-related factors [20, 28, 29].

A better understanding of factors that may affect initiation and retention in opioid agonist treatment among PWID living with HIV from both provider and PWID participant perspectives and across several countries might provide valuable insight into intervention components needed to improve OAT uptake and adherence.

HPTN 074 study evaluated an integrated intervention to facilitate HIV care and substance use treatment among PWID living with HIV in Indonesia, Ukraine, and Vietnam [30]. In HPTN 074, we conducted two rounds of qualitative interviews with study participants and healthcare providers to explore feasibility, sustainability, and strengths and weaknesses of the intervention in addressing barriers and/or enhancing facilitators among PWID living with HIV. In this paper, we present an analysis of the baseline qualitative data to describe multilevel barriers to and facilitators of OAT uptake among PWID’ and providers’ perspectives across three sites.

## Methods

### Study design

The HPTN 074 randomized controlled trial conducted in Indonesia, Ukraine, and Vietnam, assessed an intervention combining psychosocial counseling and supported referrals for ART and OAT for HIV-positive people who use drugs. The HPTN 074 study and the intervention have been described in detail elsewhere [30–32].

To evaluate the feasibility of the HPTN 074 integrated intervention, two rounds of in-depth interviews were conducted with study participants (PWID living with HIV) randomized to the intervention group and their healthcare providers (physicians and counselors/system navigators implementing the intervention) across three study sites (Kyiv, Ukraine; Thai Nguyen, Vietnam; and Jakarta, Indonesia) [31]. We present data from the first round of the qualitative interviews conducted in June 2015–March 2016, which was 1–3 months after each participant had enrolled in the trial and had completed introductory intervention sessions. As one of the intervention goals was to increase enrollment and retention in OAT, we anticipated that these interviews would highlight barriers and facilitators to OAT uptake.

### Study sites

HPTN 074 study was conducted at three study sites: Kyiv, Ukraine; Thai Nguyen, Vietnam; and Jakarta, Indonesia. These sites were chosen based on ongoing HIV epidemics among PWID, evidence of high HIV prevalence (> 25%) and/or incidence (> 4 cases/100 person-years) in PWID, according to the governmental data or ongoing studies, and also based on availability of OAT for treatment of opioid use disorders [30]. The health systems providing substance use and HIV treatment to PWID across three countries are briefly described below.

#### Indonesia

The Ministry of Health (MoH), the Ministry of Social Affair, and the Indonesia National Narcotics Board are responsible for drug treatment in Indonesia throughout outpatient (including OAT) and short-term and long-term inpatient treatment programs. The OAT methadone program reached its peak in 2009 with 2900 clients, but then decreased gradually to 2300 clients in 2015 [33]. OAT with methadone is delivered mostly at primary care settings; buprenorphine is available as a pay-for-service option in government hospitals. To access OAT, clients must be diagnosed with opioid dependence and bring a family member to help with adherence to treatment. Although the MoH provides methadone free of charge, the community health centers charge a standard OAT fee per day of Rp5000 (US$ 0.50) for clients, and hospitals charge Rp15,000 (US$ 1.60) per day to cover operational expenses [34].

Indonesia has the fourth largest number of new HIV infections per year in the world. HIV prevalence in PWID population is estimated as 39% [33]. HIV/AIDS programs are managed by the MoH; ART delivery has been scaled up and decentralized at the primary healthcare level in high HIV prevalence regions, and ART is provided free of charge [35]. However, costs for health examinations required to initiate ART are not covered by the government [31].

#### Ukraine

As of August 01, 2020, 13,701 patients were receiving OAT in Ukraine, of them 1524 (11%) patients were on buprenorphine, and the rest were on methadone [36]. Despite the gradual expansion of the OAT program, only 5.9% of estimated 350,300 of PWID in Ukraine are currently on OAT. Free-of-charge OAT is distributed mostly through narcology facilities; it is also provided at HIV and TB clinics and recently has become available in primary care facilities. (Narcology—from a Russian word “narkológija”—is a medical specialty within psychiatry dealing with diagnosis, prevention, treatment, and recovery of persons with the substance use disorders. This term has been widely used in the post-Soviet countries). To receive OAT, a person must be diagnosed with “opioid use disorder” and be registered as a drug user in the Narcology Registry. OAT is free for patients; however, to enter the program, a person must go through paid medical examinations and laboratory tests. Since 2016, patients who had been on directly observed OAT for 6 months are allowed to receive take-home doses or get OAT in pharmacies by prescription [37]. Methadone and buprenorphine can be purchased from private OAT providers.

In Ukraine, estimated HIV prevalence in PWID is 23%. Still, the coverage of PWID with ART is suboptimal: 58% of estimated number of PWID in Ukraine know their HIV-positive status, and 38% are on ART [38]. ART in Ukraine is free of charge and has been distributed only in governmental specialized healthcare facilities; it is still unavailable in the primary care. The number of co-located OAT and ART sites is increasing due to the implementation of OAT at ART sites and vice versa.

#### Vietnam

As of 2018, there were 226,860 PWID in Vietnam [39]. Since the introduction of OAT in 2008, the government of Vietnam has made great commitment to expanding the methadone program nationwide. By March 2017, over 51,000 PWID have received OAT at 280 methadone clinics across the country [40]. In order to meet the demand, private health facilities also provide OAT with methadone for PWID. While public health facilities were reported to have a better quality of treatment, private facilities succeeded in reducing the waiting time and lowering the level of stigma toward patients [40]. Buprenorphine is available only at one OAT site because of its high cost compared with methadone.

PWID receive methadone for free, but have to pay for laboratory tests before OAT initiation. In addition, as the responsibility for financing OAT program was partly shifted to provinces, OAT programs in some provinces collect fees from patients to cover costs [41]. Still, in most provinces, demand for OAT exceeds its supply [23].

Of estimated 245,337 people living with HIV in Vietnam, 131,618 persons receive ART [42]. The epidemic remains concentrated among key populations, including PWID with HIV prevalence 14%. Government of Vietnam aims at scaling up social health insurance program to achieve universal coverage with ART by 2020. To initiate ART, individuals must register in the HIV system with a support person. Since 2017, Vietnam implemented “test and start” approach for same-day ART initiation [42]. While ART is free, all mandatory blood tests must be paid for by the client [31].

### Data collection

Each site in the HPTN 074 study purposively sampled seven to ten healthcare providers for the interviews, including infectious disease and addiction physicians from HIV and addiction treatment clinics, and all study counselors/systems navigators (SNs) across all three sites, who provided intervention sessions and support to the intervention participants. In addition, each site selected 7 to 15 PWID living with HIV, of those who had been randomized to the intervention arm. PWID were recruited to the HPTN 074 by trained outreach workers through HIV testing sites, community outreach, and injection network referrals [32].

The semi-structured in-depth interviews were conducted based on a standard interview guide that was pilot tested for cultural appropriateness and used across all sites. Trained interviewers conducted 60–90-min interviews in a private room at the study site’s office, local clinic, or other convenient place. The interview guide covered the following topics: barriers to and facilitators of ART and substance use treatment and perceptions of the HPTN 074 intervention, including SN support and counseling session content. All procedures (screening, written informed consent, and interview) were conducted in the local language. Each participant received the equivalent of 8–10 USD compensation for time and travel to complete the interview. All interviews were audio-recorded, transcribed verbatim, translated into English, and imported into NVivo11 software for coding and analysis.

### Data analysis

Details of the data analysis are described elsewhere [31]. Two senior data analysts from University of North Carolina, Chapel Hill, were responsible for cross-site data analysis with support from the qualitative supervisor at each site. The senior data analysts developed a codebook with definitions of each code as well as instructions on the codes application. The codebook was organized by the main topics that were included in the interview guide. Each of the main content areas included three second-level sub-codes: informational, motivational, and financial barriers. Unanticipated barriers and facilitators which emerged in the data were subsequently included in the codebook. Study sites also added site-specific codes, to explore unique characteristics of their sites.

At each site, an experienced qualitative supervisor trained staff and supervised data collection, interviews transcription and translation, and led coding. The supervisors checked transcription and translation quality by reviewing 10% of the interviews against original audio files. Then, a team of centralized and local data coders indexed data by topics applying the codes according to the codebook. Senior data analysts checked 10% of all coded transcripts; coding differences were resolved by consensus, and code definitions were updated accordingly.

A matrix was developed to explore emergent themes and patterns around barriers and facilitators to OAT uptake [43]. Barriers and facilitators were compared across participant type (PWID vs. provider) and study sites, to identify similarities and differences in reported barriers and facilitators. Summary reports were generated and reviewed by the team.

## Results

This qualitative study sample included 62 participants: 25 healthcare providers and 37 HIV-positive PWID across the HPTN 074 study sites in three countries (Table 1).

**Table 1:** Socio-demographic characteristics of the interview participants

Characteristic	Total *n* (%)	Indonesia *n* (%)	Ukraine *n* (%)	Vietnam *n* (%)
PWID	(*n* = 37)	(*n* = 7)	(*n* = 15)	(*n* = 15)
Gender				
Male	32 (86.5)	7 (100.0)	10 (66.7)	15 (100.0)
Female	5 (13.5)	0 (0.0)	5 (33.3)	0 (0.0)
Median age (years)	35	36	33	37
Highest education completed				
Primary school	5 (13.5)	2 (28.6)	0 (0.0)	3 (20.0)
Secondary school	7 (18.9)	1 (14.3)	0 (0.0)	6 (40.0)
High school	19 (51.4)	2 (28.6)	12 (80.0)	5 (33.3)
University/College	6 (16.2)	2 (28.6)	3 (20.0)	1 (6.7)
Employment status				
Employed	18 (48.6)	3 (42.9)	6 (40.0)	9 (60.0)
Unemployed	19 (51.4)	4 (57.1)	9 (60.0)	6 (40.0)
Median length of drug use (years)	14	13	15	13
Currently on MAT	20 (54.0)	6 (85.7)	5 (33.3)	9 (60.0)

Providers	(*n* = 25)	(*n* = 10)	(*n* = 8)	(*n* = 7)
Role in clinic				
Clinician	17 (68.0)	8 (80.0)	4 (50.0)	5 (71.4)
Counselor/systems navigator	8 (32.0)	2 (20.0)	4 (50.0)	2 (28.6)
Gender				
Male	14 (56.0)	5 (50.0)	6 (75.0)	3 (42.9)
Female	11 (44.0)	5 (50.0)	2 (25.0)	4 (57.1)
Median age (years)	42.0	43.5	31.0	51.0
Highest education completed				
High School/diploma	4 (16.0)	3 (30.0)	1 (12.5)	0 (0.0)
University/college	13 (52.0)	3 (30.0)	6 (75.0)	4 (57.1)
Master/doctor/PhD	8 (32.0)	4 (40.0)	1 (12.5)	3 (42.9)
Median length of time in role (years)	5*	12	3.5	4.5*

^*^Missing: *n* = 1

Seventeen providers were clinicians, and eight were SNs. PWID were predominantly male (*n* = 32; 86.5%); female PWID were represented only in Ukraine (5 of 15 participants or 33%), which reflected PWID gender distribution at all three sites [31]. The median age of PWID was 35 years; they were slightly younger in Ukraine and older in Vietnam. On average, over half were unemployed.

### Barriers to OAT uptake by PWID living with HIV

Overall, PWID and their healthcare providers across all sites reported numerous, similar barriers to OAT initiation, although there were some country-specific differences (Table 2).

**Table 2:** Barriers to substance use treatment: key themes

Themes	PWID	Providers
Structural level		
Complicated entry to MAT program	All sites: limited number of treatment slots; waiting lists Vietnam and Indonesia: procedural barriers; strict admission requirements	Vietnam: low capacity of MAT sites to accept new patients Indonesia, Vietnam: strict admission requirements to start MAT
Problematic clinic access	Majority in Vietnam, some in Indonesia: long distance to MAT clinic Ukraine, Vietnam: inflexible clinic hours; lines at MAT sites	All sites: long distance to MAT clinic; limited/inflexible clinic hours Indonesia, Ukraine: daily visits to MAT site
Financial barriers	Costly examinations to start MAT (Indonesia, Ukraine); a need to pay for transportation and supporting services at MAT site (Vietnam)	Most providers in Indonesia and a few in Ukraine and Vietnam: costly examinations to start MAT Vietnam: a need to pay for MAT leads to patients skipping doses
Community level		
Social stigma toward PWID	All sites: stigma toward PWID in the community rather than at health facilities Vietnam: social stigma toward methadone clients	Indonesia, Ukraine: social stigma toward PWID Ukraine: stigmatization of addiction treatment per se; negative image of narcology institutions
Individual level		
Lack of information about substance use treatment	Indonesia, Ukraine: lack of information about available substance use treatment	All sites: PWID’ lack of information about available substance use treatment; lack of understanding of addiction and MAT in society
Negative opinion of methadone treatment	All sites—misconceptions of methadone: it is “drug given for free” (Ukraine, Vietnam) and “worse than street drugs” (Indonesia, Vietnam) Ukraine: PWID would prefer buprenorphine	Ukraine: misconceptions and negative opinions of methadone among PWID; PWID would prefer buprenorphine Some providers see MAT as a free substitution to a street drug
Other barriers related to drug use	Ukraine: most PWID are used to the drug user’s lifestyle Ukraine, Vietnam: using other substances when on MAT as a barrier to adherence	Ukraine: PWID do not start MAT as they prefer a “drug user’s life” and demonstrate “lack of will” (lack of internal motivation)
Drug interactions	Ukraine, Vietnam: fear of ART and methadone interaction	

#### Complicated entry to OAT program

At all three sites, PWID talked about complicated entry to OAT, combined with a limited number of available treatment slots, and waiting lists to start OAT.

*Interviewer (I): How long do the PWID have to wait until they get methadone?*

*Respondent (R): In case of waiting list, just wait until someone dies. Then the next drug user can automatically fill the vacant position…*

*I: How long did you wait?*

*R: About 7 months until they called me.*

*I: Did that mean some methadone client had died?*

*R: Yes, I guess so* ***(PWID, male, 36 y.o., Indonesia).***

In Vietnam, PWID reported multiple procedural barriers and strict admission requirements: local community quotas to enroll in OAT—“*only 4–5 persons every year*” ***(PWID, male, 37 y.o., Vietnam)***, need to wait for authorities’ approval of one’s application for OAT, and family presence required for registration at OAT site, which was also the case in Indonesia. According to the respondents, some PWID may want to start OAT, but are unable to meet requirements, not having relatives to accompany them to the OAT site.

Similar to the PWID, most providers in Indonesia and Vietnam described admission requirements: bureaucracy and tedious paperwork, required presence of a family member, and the need to obtain the local authorities’ approval.

*I: Which step is the most difficult?*

*R: The first step, meeting administrative requirements, because not everyone has an ID card and not everyone has a family member. Usually the junkies have already been disowned by their families, and the families do not want to know about their condition anymore* ***(counselor/SN, female, Indonesia).***

However, according to a provider in Vietnam, the admission procedure was simplified recently, which ironically led to a problem with site capacity mentioned by PWID: OAT facilities have insufficient number of treatment slots to accommodate all the PWID seeking program entry.

*The demand on OAT is very high. 270 patients are in the clinic now, exceeding the possible threshold of 150 patients; and the demand for treatment is still high. Current instruction prohibits to receive more patients because it is over the capacity limit to provide services* ***(SN/physician at ART clinic, Vietnam).***

#### Problematic clinic access

Clinic inaccessibility was a persistent theme in PWID and provider accounts across all sites. Most PWID in Vietnam and some in Indonesia reported daily long trips by motorbike or public transport to their OAT clinic—“*about three times transport change”* (***PWID, male, 37 y.o., Indonesia***)—as a huge barrier to clinic access. In addition, PWID in Ukraine and Vietnam talked about inflexible clinic hours, conflict with their working hours, and lines at OAT sites. Many PWID reported that OAT interfered with holding a full-time job.

*I can only work on some minor jobs. I spend all the time in the morning for this [OAT], only afternoon is left. It is difficult—someone hires me to do some job nearby, then I try to take time to go; basically, I cannot do any job* (***PWID, male, 36 y.o., Vietnam***).

Providers across all sites talked about the same structural barriers to initiation and retention in OAT. They reiterated PWID concerns about the need to visit the OAT site daily, its inconvenient location, and inflexible clinic hours.

*OAT should be accessible, literally. A man from Vinogradar shouldn't have to go to somewhere in Svyatoshin—he should come to the clinic near his home and get his pills there, both ART and OAT. Going somewhere, you spend time and money. It all should be close to your place—same as a kindergarten or a school, OAT clinic should be nearby* ***(counselor/SN, male, Ukraine)***.

#### Financial barriers

Most PWID at all sites reported financial burden related with OAT initiation: costly procedures to enter OAT (numerous mandatory examinations) in Indonesia and Ukraine, costly medication (buprenorphine) in Indonesia and Vietnam, and a need to pay for transportation to the clinic and supporting services on-site (parking, cups, tests) in Vietnam.

*I: Do you have to pay for Suboxone and the doctor? How much?*

*R: I pay 100,000 for the doctor, to buy the drugs, 50,000 per strip, Riclona 100,000 per strip, alprazolam 50,000 per strip… I should pay the doctor, then should buy the medicines. I am not a rich person, why don’t I get a net price, not to bear this much! If the goal is to quit drugs, I don’t think this is the way, because my friend can buy the drugs cheaper…* ***(PWID, male, 23 y.o., Indonesia).***

Most providers in Indonesia and some in Ukraine and Vietnam also referred to costly procedures to start OAT (mandatory laboratory tests and other examinations) as a barrier for PWID who are often unemployed and have limited financial resources. In Vietnam, where patients or their families have to pay for methadone prescription, providers believed that such financial burden makes patients skip the doses, sometimes for weeks.

#### Social stigma toward PWID

Across all sites, PWID talked about stigma and social devaluation of people who use drugs; in their opinion, such stigma was more common in community than at healthcare facilities. In addition, according to PWID in Indonesia and Vietnam, people in community do not differentiate between active drug users and OAT patients, so joining OAT means that you confirm that you are “drug user.” Similarly, providers in Indonesia and Ukraine reported social stigma toward both PWID and addiction treatment.

*It turns out that if you are a drug addict, then in any case you are a thief, a villain, or something like that…* (***PWID, male, 33 y.o., Ukraine***)

Unlike other participants, one PWID in Vietnam recognized that people in his community were very supportive of his OAT initiation, “*Everyone is happy for me, they come and talk with me*” (***PWID, male, 36 y.o., Vietnam***). Such support motivated this person to retain in treatment.

#### Lack of information about OAT

Some PWID in Indonesia and Ukraine mentioned lack of information on available substance use treatment, as well as lack of general understanding of OAT.

*I: What methods of available substance use treatment do you know?*

*R: To be honest, I’ve been injecting for such a long time, I am supposed to know everything, and in the end I do not know anything. Well, I know that there is a detox, but maybe I cannot quite understand what it is…****(PWID, female, 35 y.o., Ukraine) ***.

Providers across all sites saw the clients’ lack of information about available treatment as a barrier to OAT initiation; they also noted general lack of understanding of addiction and  OAT in society. A provider from Ukraine shared his concerns about negative image of addiction treatment institutions, rooted in the Soviet era.

Of course, for many of them [PWID], it is very difficult to make a decision, because they do not know anything about available range of services they could get. For many, the image of drug treatment clinic since Soviet times is some punitive institution, where he will be tied to a bed and experience some incredible tortures…*** (OAT physician, male, Ukraine)***.

#### Negative opinion of methadone treatment

PWID across all sites expressed negative opinion of OAT and specifically of treatment with methadone. Such opinion was overwhelmingly pronounced in Ukraine where PWID associated methadone with lack of freedom and lifelong treatment. Many Ukrainian and Vietnamese PWID considered methadone a free drug rather than medication—“*they substitute one drug with another*” ***(PWID, male, 39 y.o., Ukraine).*** Others, especially in Indonesia and Vietnam, believed that “*it is better to use drugs than methadone*” ***(PWID, male, 39 y.o., Vietnam)***. Most PWID in Ukraine would prefer buprenorphine to methadone due to beliefs that buprenorphine is less toxic and that quitting methadone was impossible once you started it.

*OAT is like a double-edged sword. Some people think that OAT was invented to simply eliminate injecting drug users… A person who uses methadone for some time, especially methadone,—he turns into a vegetable, especially with high dosage. He only goes to OAT and back home, nothing else…****(PWID, male, 33 y.o., Ukraine)***

Providers in Ukraine confirmed that PWID had misconceptions about methadone; they cited their patients who perceived methadone treatment as “*chemicals that destroy my body*” ***(counselor/SN, female, Ukraine)***, “*point of no return” *and having “*one foot in a grave*” ***(counselor/SN, male, Ukraine***). Both physicians and counselors in Ukraine believed that many PWID would join OAT if free buprenorphine was available.

#### Other barriers related to drug use

Ukrainian PWID often talked about their drug dependence and drug use, which takes up all their time, saying they were so accustomed to a drug user’s life. PWID in Ukraine and Vietnam also mentioned using other substances when on OAT, as a barrier to adherence.

*I: So you took methadone for two years and left the program. Why do people quit OAT, what are their reasons?*

*R: Basically, I would be happy not to use drugs, but 20 years of use—well, I've already forgotten how it is, (to live) without drugs…* ***(PWID, male, 37 y.o., Ukraine)***

*They quit because they still “play” with drugs, and they think that using both drug and medication, they are not going to have craving anymore, but actually taking both, it is even more craving. It fights against each other; therefore, they have to quit methadone—it is better to use only drugs* ***(PWID, male, 39 y.o., Vietnam)***

In Ukraine, providers saw drug use-related barriers somewhat differently: they believed that PWID “*cannot imagine life without drugs*” because they “*want to be under the influence*” ***(counselor/SN, male, Ukraine).*** For them, drug dependence and lack of motivation for treatment were equivalent. Providers (but not PWID) in Ukraine felt that some PWID might not start addiction treatment because of “*lack of will*” (***ID physician, female, Ukraine***) and laziness; they perceived that PWID prioritized drug use over caring for their health and hence were less motivated to take up ART and OAT. In providers’ opinion, PWID would initiate OAT only in a critical situation, “*When they are broke and have no money for the drugs, then they come to us*.” ***(OAT physician, male, Ukraine).***

#### Fear of drug interactions

As the study participants were PWID living with HIV, some in Ukraine and Vietnam who were on ART explained their reluctance to start OAT by fear of interaction between ART and methadone.

*R: While taking methadone, also taking ART, the medication is resistant.*

*I: What does it mean?*

*R: For example, I take the dose of 100 mg; it is reduced to 50 only* ***(PWID, male, 42 y.o., Vietnam)***

### Facilitators

PWID and providers across all sites reported far fewer facilitators than barriers to substance use treatment uptake (Table 3). As in the case of barriers, PWID and providers across study sites described similar facilitators to initiate OAT.

**Table 3:** Facilitators to substance use treatment: key themes

Themes	PWID	Providers
Individual level		
Internal motivation for a life change	Indonesia, Ukraine: being tired of using drugs; a will for a life change	Ukraine, Vietnam: being tired of a drug user’s life; a will for a life change
Interpersonal level		
Social support	All sites: MAT information/motivation from peers and providers Indonesia, Vietnam: support from peers; support (Indonesia) and pressure (Ukraine) from the family	MAT information/motivation from peers and providers (Indonesia and Vietnam) and from local HIV-servicing CBOs (Ukraine) All sites: family support of MAT initiation and adherence

#### Internal motivation for a life change

“*Internal motivation*” for a life change was the main facilitating factor for quitting drug use and starting OAT reported by PWID in Indonesia and Ukraine; they felt tired of drug use and expressed a will “*to live a normal life*” ***(PWID, male, 38 y.o., Indonesia)*** without drugs. Similarly, providers in Ukraine and Vietnam spoke about PWID being tired of a drug user’s life, considering their internal will for a life change as a motivator to OAT initiation.

*I: What was your personal reason to start OAT?*

*R: First, I was already tired of such a life that I had (laughs). It is in the first place. I already wanted to change it, make it at least a little better. Plus, I want to have kids, I want to live a normal life. Not to exist, but to live a life* ***(PWID, male, 42 y.o., Vietnam).***

#### Social support

According to PWID across all sites, social support and particularly OAT information and motivation from peers and providers facilitated their treatment uptake. Support and opinion of friends/peers were important for PWID in Indonesia and Vietnam, as well as support (Indonesia) and even pressure (Ukraine) from the family.

*In my case, a pregnancy of my wife drove me to the drug treatment program. Also, I was curious about benefit of methadone because I heard a little from my friends. Finally, my family encouraged me to join [OAT]. I felt guilty looking at my wife and child who did not eat sufficiently. When I joined methadone, I realized that I could earn legal money for them**** (PWID, male, 36 y.o., Indonesia)***

Similarly, providers in Indonesia and Vietnam believed that OAT information and motivation from peers and providers were helpful, as well as information provided by local community-based organizations in Ukraine. Across all sites, providers considered the family influence important for PWID engagement in OAT.

## Discussion

We explored factors that influence OAT uptake among PWID in Jakarta, Indonesia; Kyiv, Ukraine; and Thai Nguyen, Vietnam. The unique features of this study are that our participants were PWID living with HIV, the compared opinions between PWID (HPTN 074 participants) and healthcare providers, and the cross-cultural nature of this study. We found that despite different cultures and healthcare systems, there was a significant overlap of reported barriers and facilitators to engagement in OAT across the study countries.

HIV-positive status has a significant impact on access to drug treatment services in many countries. In Ukraine, the proportion of HIV-positive patients on OAT is 55%, whereas HIV prevalence in the overall PWID population is 23% [36]. Similar discrepancy is present in Vietnam and Indonesia. This is explained by the fact that HIV-positive PWID were prioritized in OAT scale-up. Nevertheless, as we found in our study, HIV-positive PWID continue to have significant and distinct barriers to OAT.

Overall, both PWID and healthcare providers at all sites reported similar structural barriers to OAT initiation and retention. Among them, complicated entry to OAT program (limited treatment slots, complicated admission requirements) and problematic clinic access (distance to the clinics, inflexible clinic hours) were repeatedly mentioned. To improve OAT accessibility, it is necessary to set flexible inclusion criteria to ensure PWID immediate access to treatment and eliminate waiting lists [44]. Previously, it has been documented that rigid control associated with OAT delivery is a predictor of treatment interruption [45]. In addition, restrictive OAT practices obstruct the improvement of social functioning of OAT patients and their return to a desired “normal life” [46]. Given the vast access barriers in Vietnam and Indonesia, policymakers might look for alternative approaches to current drug treatment models in the countries with limited resources, rather than translating the models for developed countries. Low-threshold OAT services (geographical accessibility, patient-friendly clinic hours, and flexible models of OAT distribution), reported as facilitators by providers in our study, are strongly recommended to encourage drug treatment entry and retention.

Financial barriers to OAT were apparent at all sites, related both to OAT initiation and maintenance, but there may have been financial barriers of a different intensity. For instance, in Indonesia and Ukraine, PWID talked about costly examinations and required donations to enter OAT, while transportation to OAT site entailed specific expenses in Vietnam and Indonesia. It was shown in our study and elsewhere [46] that long daily trips and rigid clinic hours prevent OAT patients from holding a stable job. For OAT sites, one recommendation is to develop a checklist to ask clients about potential financial barriers and to develop strategies to address these barriers. On the structural level, expanding prescription OAT and take-home doses, which are a known predictor of retention in OAT programs [28], is highly recommended for stabilized patients. This will enable clinics to serve more individuals, to address the problems of long and costly transportation and of incompatibility of OAT with employment and other meaningful activities [45]. Availability of take-home doses is recommended for all countries to diminish patients’ financial burden of everyday trips to the clinic, as well as to ensure higher treatment retention.

Insufficient coverage with OAT in all three countries is a pressing issue. Decentralization of OAT services and their integration in primary care, as well as its availability at HIV and TB treatment sites, are another potential solution.

Social stigma toward PWID (including OAT patients) reported by both PWID and providers in our study had been previously described in the study countries (Vietnam, Ukraine) and beyond [23, 47]. Similar to our findings, in Vietnam, Tran et al. [23] found that stigma toward PWID was significantly higher in the community than at healthcare settings.

A significant body of research has shown that key populations disproportionately affected by the HIV epidemic may experience multiple stigmas, related to HIV and to other characteristics or behaviors such as substance use, sex work, and incarceration. Such intersecting stigmas have been associated with a range of vulnerabilities and risks [48, 49]; they may exacerbate one another and have complex effects on health behaviors and health outcomes [48, 50]. While recent study from Ukraine demonstrated much better outcomes at each stage of the HIV care cascade among those OAT patients who received integrated OAT and ART services [51], another study has shown that even at the sites with co-located HIV and drug treatment services OAT patients may experience high levels of HIV-related and substance use stigmas [52].

Our data indicate the need for informational campaigns and community-level interventions to change societal attitudes and counter stigma associated with addiction and OAT (especially with methadone) in all three countries and to ensure community support for those PWID who plan to initiate OAT.

Individual-level barriers reported by the study participants included PWID’ lack of information on existing OAT services, drug use-related barriers to health care, and prejudices and negative attitudes to OAT, especially to treatment with methadone.

In the study of the interdependence of the barriers to OAT among PWID who had never received OAT in Ukraine, Zelenev et al. [53] concluded that in the hierarchy of barriers, the perceptions about OAT efficacy and its negative impact on health were the most widespread, followed by structural barriers and social stigma. In our study and elsewhere [46], limited knowledge and negative attitudes to methadone therapy have been reported as significant obstacles for OAT initiation and retention among study participants. Such negative attitudes may cause another financial burden by buprenorphine preferred by many PWID, as methadone is free for patients in all countries. Because PWID’ positive or negative attitudes and prejudices around OAT might be reinforced through their social interactions, utilizing drug users’ networks to deliver information about OAT may be an effective strategy to address both misconceptions of methadone and lack of information about drug treatment services. OAT patients could be trained to effectively communicate the value of OAT in their social circle and address myths surrounding drug treatment. Such peer-delivered interventions could also ensure social support shown in our study to be one of the main facilitators for substance use treatment.

As for many participants unavailability of the preferable OAT medication constituted a barrier to treatment access, to increase coverage of PWID with OAT, it is important to take into account patients' choices and preferences, at the same time paying attention to debunking myths and misconceptions.

Patient and provider communication about OAT treatment goals may reduce patient frustration, uncertainty, and fears of “lifelong treatment.” In our study, PWID did not mention their problematic relationship with providers. However, lack of realistic information about drug treatment even in PWID with OAT experience shows evident gaps in patient–provider communication. Improving such communication is important to provide social support to the patients, debunk the myth of the dangers of methadone, explain drug interaction, and ensure that methadone dosage is sufficient, especially for people on ART.

One strength of our study is that it shows unique barriers inherent to PWID living with HIV, such as fear of ART and OAT interaction. Information about drug interaction and side effects should be a part of health promotion interventions for PWID in the countries with high HIV prevalence in PWID. In addition, these findings emphasize the need for such interventions to be tailored to the specific needs of PWID living with HIV, who might face challenges in taking both therapies.

While most individual and structural barriers were reported by both PWID and providers, there were some differences: PWID talked about fear of drug (OAT and ART) interactions, while providers emphasized PWID prioritization of drug use over caring about health. At the same time, different from the findings of other studies in Ukraine [19, 54, 55], in our study neither PWID nor providers mentioned police violence or fear of official registration at Narcology Registry as a barrier to OAT uptake. Similarly, participants did not mention OAT doses as a barrier to treatment. These omissions may be related to changes in legislation regarding dosage and with police reform in the country.

Social support and motivation for a life change were recognized by both PWID and providers as facilitators to OAT uptake. Given that substantial barriers for OAT treatment entry and retention were observed in all three countries, social support from family and healthcare providers may help to maintain drug treatment motivation when faced with treatment barriers. The HPTN 074 experimental intervention included a session with “a supporter” to strengthen participants’ family support, but was focused on ART initiation. As we found that family support and family well-being were among the main motivators for the life change, this approach could be expanded to substance use treatment. However, as many potential supporters may lack basic information about drug dependence, such programs should also include an educational component.

To embed OAT in their lives, PWID must believe that such treatment would be effective rather than harmful. There is a considerable need to improve the image of OAT programs by introducing effective social marketing campaigns for PWID and community. Still, future research is needed on potential sources of support for OAT within the social networks of PWID and to identify trustworthy and reliable sources of information on substance use treatment. Public health professionals and policymakers need to be aware of the multilevel barriers to OAT experienced by PWID. Being unaware of the documented barriers, these stakeholders may continue attribute OAT dropout to lack of motivation and hence fail to address the critical obstacles to OAT. OAT providers should also examine how program requirements may add additional barriers that cause challenges, especially for impoverished individuals, many of whom are in poor health condition.

### Limitations

As described, the structure of drug treatment varies between the countries; hence, different approaches may be needed to achieve similar solutions. While the multi-site nature of this qualitative study is one of its strengths, it limits making general conclusions and recommendations. In addition, data were collected in multiple languages and then translated into English for analysis [31]. Although quality assurance/quality control procedures were used to minimize translation errors, some quality of the transcripts could be lost in translation. At the same time, as the interview guide was standardized and pilot tested for cultural appropriateness across all sites, our approach allowed different patterns to emerge allowing for within- and cross-country comparisons.

## Conclusions

Overall, both PWID and providers reported similar and multilevel barriers to OAT uptake in all three countries where insufficient coverage with OAT is a pressing issue.

While we aimed to explore barriers to OAT initiation, PWID participants spoke about barriers that PWID might face both prior to, and also during, their OAT treatment, such as distance to OAT site, inability to maintain a job, expenses at OAT site, and stigma in community. These results highlight a need for support for PWID at each stage of the drug treatment, including both initiation and retention in care. To expand OAT, it is necessary to ensure low-threshold, financially affordable OAT programs.

Negative personal beliefs and attitudes, coupled with structural barriers to OAT uptake, indicate a need for a review of existing practices, development of novel interventions for PWID, and delivery of marketing campaigns that can counter misinformation in community associated with drug dependence and OAT in all study countries. Future research should examine the sources and perceived trustworthiness of information on OAT from social media, peers, and social marketing. This information could be used to develop programs to not only promote OAT but also to address the negative misinformation about OAT. Furnishing drug treatment programs with feedback about barriers to drug treatment may help facilitate organizational problem solving to address these barriers.

## Data Availability

The data collected and analyzed during this study are not publicly available due to confidentiality reasons. However, de-identified transcripts might be available from the corresponding author and PI of the study (William C. Miller) upon reasonable request.

## Abbreviations

OAT: Opioid agonist treatment
HIV: Human immunodeficiency virus
PWID: People who inject drugs
ART: Antiretroviral therapy
NSP: Needle and syringe program
MoH: Ministry of Health
SN: System navigator
